# Supplementary material for: KIF16B drives MT1-MMP recycling in macrophages and promotes co-invasion of cancer cells
Source: Life Sci Alliance. 2023 Sep 11;6(11):e202302158. doi: 10.26508/lsa.202302158 (PMC10494930; doi:10.26508/lsa.202302158)
Supplement: Supplementary file 4 [file LSA-2023-02158_TableS1.docx]

| **Figure 1** | |
| --- | --- |
|  | |
| **Figure 1 H** | |
| **condition** | **relative co-localization to MT1-MMP-mCherry [%] ± SEM** |
| KIF16B-YFP | **55.66 ± 1.68** |
| KIF16B | **20.66 ± 1.44** |
| kinesin-1 | **4.37 ± 0.57** |
| kinesin-2 | **2.01 ± 0.35** |
|  | |
| **Figure 1 I** | |
| **marker** | **mean co-localization to KIF16B-YFP positive vesicles [%]**  **± SEM** |
| Rab 5 | **24.25 ± 3.02** |
| Rab 7 | **11.71 ± 4.73** |
| Rab8 | **21.38 ± 4.94** |
| Rab 14 | **48.27 ± 5.98** |
| Rab 22a | **4.89 ± 1.97** |
|  | |
| **Figure 1 J** | |
| **marker** | **mean co-localization to KIF16B-YFP/MT1-MMP-mCherry positive vesicles [%] ± SEM** |
| Rab 5 | **23.82 ± 2.75** |
| Rab 7 | **6.66 ± 2.33** |
| Rab8 | **6.60 ± 2.18** |
| Rab 14 | **26.84 ± 1.59** |
| Rab 22a | **19.03 ± 2.61** |
|  | |

| **Figure 2** | | | | | | | | | | | |
| --- | --- | --- | --- | --- | --- | --- | --- | --- | --- | --- | --- |
|  | | | | | | | | | | | |
| **Figure 2 B** | | | | | | | | | | | |
|  | | **expression [%]** | | | | | | | | | |
| **condition** | | **donor I** | | | **donor II** | | | **donor III** | | | **mean ± SEM** |
| ctrl | | 100 | | | 100 | | | 100 | | | **100 ± 0** |
| KIF16B #1 | | 40.87 | | | 34.14 | | | 36.72 | | | **37.24 ± 1.96** |
| KIF16B #2 | | 14.97 | | | 24.97 | | | 25.79 | | | **21.91 ± 3.48** |
| KIF16B #3 | | 17.80 | | | 26.43 | | | 44.22 | | | **29.48 ± 7.78** |
|  | | | | | | | | | | | |
| **Figure 2 D** | | | | | | | | | | | |
|  | | **fluorescence intensity [%]** | | | | | | | | | |
| **condition** | | **donor I** | | | **donor II** | | | **donor III** | | | **mean ± SEM** |
| ctrl | | 122.98 | | | 89.01 | | | 88.01 | | | **100 ± 11.49** |
| KIF16B #1 | | 39.49 | | | 26.83 | | | 38.21 | | | **34.84 ± 4.03** |
| KIF16B #3 | | 40.46 | | | 31.6 | | | 31.87 | | | **34.64 ± 2.91** |
| Rab14 | | 30.25 | | | 29.20 | | | 34.86 | | | **31.44 ± 1.74** |
| KIF16B #1 + Rab14 | | 22.1 | | | 24.33 | | | 39.02 | | | **28.48 ± 5.31** |
| KIF16B #3 + Rab14 | | 32.79 | | | 24.81 | | | 27.61 | | | **28.40 ± 2.34** |
| statistical analysis: unpaired t-test | | | | | | | | | | | |
| **Figure 2 F** | | | | | | | | | | | |
|  | **surface MT1-MMP [%]** | | | | | | | | | | |
| **condition** | **donor I** | | **donor II** | | | **Donor III** | **donor IV** | | | **mean ± SEM** | |
| ctrl | 100 | | 100 | | | 100 | 100 | | | **100 ± 0** | |
| KIF16B #1 | 75.13 | | 55.48 | | | 76.77 |  | | | **69.13 ± 6.84** | |
| KIF16B #2 | 47.18 | | 66.45 | | | 68.73 | 63.73 | | | **61.42 ± 4.87** | |
| Rab14 | 57.52 | | 62.73 | | | 87.01 | 47.46 | | | **63.68 ± 8.40** | |
| KIF16B #2 + Rab 14 | 49.46 | | 44.36 | | | 47.72 |  | | | **47.18± 1.50** | |
| statistical analysis: one-sample t-test | | | | | | | | | | | |
| **Figure 2 H** | | | | | | | | | | | |
|  | **surface MT1-MMP [%]** | | | | | | | | | | |
| **condition** | **donor I** | | **donor II** | | | **donor III** | **donor IV** | | | **mean ± SEM** | |
| ctrl | 100 | | 100 | | | 100 | 100 | | | **100 ± 0** | |
| KIF16B #1 | 77.76 | | 63.43 | | | 70.58 |  | | | **70.59 ± 4.14** | |
| KIF16B #2 | 30.61 | | 77.94 | | | 45.96 | 56.78 | | | **52.82 ± 9.95** | |
| KIF16B #2 + Rab14 | 71.7 | | 62.87 | | | 79.68 | 36.46 | | | **62.68 ± 9.39** | |
| statistical analysis: one-sample t-test | | | | | | | | | | | |
| **Figure 2 J** | | | | | | | | | | | |
|  | | | | **siRNA** | | | | | | | |
|  |  |  |  | **ctrl** | | | | | **KIF16B #2** | | |
| donor I | | | | 100 | | | | | 44.67 | | |
| donor II | | | | 100 | | | | | 1.83 | | |
| **total** | | | | **100** | | | | | **23.25 ± 21.42** | | |
|  | | | | | | | | | | | |

| **Figure 3** | | | | | | | | | |
| --- | --- | --- | --- | --- | --- | --- | --- | --- | --- |
|  | | | | | | | | | |
| **Figure 3 G** | | | | | | | | | |
| **overexpression** | | | | | **mean ± SEM** | | | | |
| MT1-MMP mCherry | EYFP | | | | **131 ± 6.90** | | | | |
|  | KIF16B-YFP | | | | **130.7 ± 7.41** | | | | |
|  | KIF16B-Cterm-YFP | | | | **72.65 ± 5.46** | | | | |
| statistical analysis: unpaired t-test | | | | | | | | | |
| **Figure 3 J** | | | | | | | | | |
|  | | **DMSO treated cells showing vesicular/dispersed phenotype** | | | | | | | |
| **phenotype** | | **donor I** | | **donor II** | | **donor III** | | **mean ± SEM** | |
| vesicular | | 23 | | 31 | | 31 | | **28.33 ± 2.67** | |
| dispersed | | 11 | | 7 | | 4 | | **7.33 ± 2.03** | |
| statistical analysis: paired t-test | | | | | | | | | |
| **Figure 3 K** | | | | | | | | | |
|  | | **wortmannin treated cells showing vesicular/dispersed phenotype** | | | | | | |  |
| **phenotype** | | **donor I** | | **donor II** | | **donor III** | | **mean ± SEM** |  |
| vesicular | | 0 | | 0 | | 6 | | **2 ± 2.00** |  |
| dispersed | | 42 | | 33 | | 29 | | **34.67 ± 3.84** |  |
| statistical analysis: paired t-test | | | | | | | | |  |
| **Figure 3 N** | | | | | | | | |  |
|  | | | **KIF16B-YFP vesicle number/cell ± SEM** | | | | | |  |
|  |  |  | **DMSO** | | | | **wortmannin** | |  |
| donor I (n = 10; 11) | | | 74.5 ± 16.38 | | | | 12.36 ± 0.96 | |  |
| donor II (n = 10) | | | 65.1 ± 13.91 | | | | 24.8 ± 7.19 | |  |
| donor III (n = 10) | | | 51.6 ± 4.98 | | | | 18.5 ± 3.34 | |  |
| **total** | | | **63.73 ± 7.305** | | | | **18.35 ± 2.659** | |  |
| statistical analysis: unpaired t-test | | | | | | | | |  |
| **Figure 3 O** | | | | | | | | |  |
|  | | | **KIF16B-YFP vesicle size [µm^2^] ± SEM** | | | | | |  |
|  |  |  | **DMSO** | | | | **wortmannin** | |  |
| donor I (n = 10; 11) | | | 0.13 ± 0.03 | | | | **0.22** ± 0.04 | |  |
| donor II (n = 10) | | | 0.1 ± 0.02 | | | | **0.22** ± 0.03 | |  |
| donor III (n = 10) | | | 0.08 ± 0.01 | | | | **0.24** ± 0.02 | |  |
| **total** | | | **0.105 ± 0.0109** | | | | **0.227 ± 0.22** | |  |
| statistical analysis: unpaired t-test | | | | | | | | |  |

| **Figure 4** | | | | | | | | | | | | | |
| --- | --- | --- | --- | --- | --- | --- | --- | --- | --- | --- | --- | --- | --- |
|  | | | | | | | | | | | | | |
| **Figure 4 E** | | | | | | | | | | | | | |
|  | | **degradation [%]** | | | | | | | | | | | |
| **condition** | | **donor I** | | | **donor II** | | | **donor III** | | | | **mean ± SEM** | |
| ctrl | | 87.57 | | | 124.36 | | | 88.07 | | | | **100 ± 12.18** | |
| KIF16B #1 | | 52.54 | | | 67.02 | | | 59.81 | | | | **59.79 ± 4.18** | |
| KIF16B #3 | | 37.22 | | | 69.24 | | | 55.85 | | | | **54.10 ± 9.29** | |
| Rab14 | | 35.85 | | | 78.09 | | | 39.98 | | | | **51.31 ± 13.44** | |
| KIF16B #1 + Rab14 | | 26.83 | | | 54.58 | | | 37.06 | | | | **39.49 ± 8.10** | |
| KIF16B #3 + Rab14 | | 32.02 | | | 50.75 | | | 38.40 | | | | **40.39 ± 5.50** | |
| statistical analysis: unpaired t-test | | | | | | | | | | | | | |
| **Figure 4 F** | | | | | | | | | | | | | |
|  | | **podosomes / cell [%]** | | | | | | | | | | | |
| **condition** | | **donor I** | | | **donor II** | | | **donor III** | | | | **mean ± SEM** | |
| ctrl | | 87.79 | | | 84.21 | | | 128.00 | | | | **100 ± 14.04** | |
| KIF16B #1 | | 95.93 | | | 98.71 | | | 109.23 | | | | **101.29 ± 4.05** | |
| KIF16B #3 | | 92.10 | | | 79.99 | | | 102.23 | | | | **91.44 ± 6.43** | |
| Rab14 | | 95.63 | | | 84.78 | | | 125.14 | | | | **101.85 ± 12.06** | |
| KIF16B #1 + Rab14 | | 97.28 | | | 78.36 | | | 97.77 | | | | **91.14 ± 6.39** | |
| KIF16B #3 + Rab14 | | 96.65 | | | 108.34 | | | 113.69 | | | | **106.23 ± 5.03** | |
| statistical analysis: unpaired t-test | | | | | | | | | | | | | |
| **Figure 4 G/H** | | | | | | | | | | | | | |
|  | | **mean number of cells [%]** | | | | | | | | | | | |
| **podosome number:** | | **0-100** | | **101-200** | | **201-300** | | | **301-400** | **401-500** | | | **>500** |
| ctrl | | 21.84 | | 26.44 | | 18.39 | | | 17.24 | 11.49 | | | 4.60 |
| KIF16B #1 | | 13.79 | | 34.48 | | 25.29 | | | 13.79 | 6.90 | | | 5.75 |
| KIF16B #3 | | 24.14 | | 26.44 | | 27.59 | | | 14.94 | 4.60 | | | 2.30 |
|  | | | | | | | | | | | | | |
| **Figure 4 K** | | | | | | | | | | | | | |
|  | | **invading cells [%]** | | | | | | | | | | | |
| **condition** | | **donor I** | | | **donor II** | | | **donor III** | | | | **mean ± SEM** | |
| ctrl | | 100 | | | 100 | | | 100 | | | | **100 ± 0** | |
| KIF16B #1 | | 61.05 | | | 58.77 | | | 68.98 | | | | **62.94 ± 3.10** | |
| KIF16B #3 | | 62.01 | | | 52.93 | | | 54.35 | | | | **56.43 ± 2.82** | |
| statistical analysis: one-sample t-test | | | | | | | | | | | | | |
| **Figure 4 L** | | | | | | | | | | | | | |
|  | | | **distance of invasion [pixels] ± SEM** | | | | | | | | | | |
|  | **FOV ea.** | | **ctrl** | | | | **KIF16B #1** | | | | **KIF16B #3** | | |
| donor I | 10 | | 301.48 ± 15 | | | | 223.22 ± 16.39 | | | | 211.91 ± 14.3 | | |
| donor II | 10 | | 440.43 ± 13.63 | | | | 331.45 ± 19.15 | | | | 322.84 ± 12.23 | | |
| donor III | 10 | | 309.81 ± 17.97 | | | | 269.87 ± 15.95 | | | | 265.58 ± 14.71 | | |
| **total** | **90** | | **350.6 ± 14.68** | | | | **274.8 ± 12.64** | | | | **266.8 ± 11.39** | | |
| statistical analysis: unpaired t-test | | | | | | | | | | | | | |

| **Figure 5** | | | |
| --- | --- | --- | --- |
|  | | | |
| **Figure 5 I** | | | |
| **day** | **condition** | **spheroids** | **mean spheroid area ± SEM** |
| 0 | ctrl | **25** | **347,473 ± 5,479** |
|  | KIF16B #2 | **21** | **336,646 ± 9,556** |
| 3 | ctrl | **24** | **531,446 ± 21,680** |
|  | KIF16B #2 | **25** | **541,965 ± 25,380** |
| statistical analysis: unpaired t-test | | | |
| **Figure 5 J** | | | |
| **day** | **condition** | **spheroids** | **mean detected particle number ± SEM** |
| 3 | ctrl | **27** | **95.7 ± 9.58** |
| 3 | KIF16B #2 | **21** | **68.33 ± 6.17** |
| statistical analysis: unpaired t-test | | | |

| **Supplementary Figure 2** | | | | | |
| --- | --- | --- | --- | --- | --- |
|  | | | | | |
| **Supplementary Figure 2 E** | | | | | |
|  | **Endogenous KIF16B vesicle size [µm^2^] ± SEM** | | | | |
|  | **DMSO** | | | **wortmannin** | |
| donor I (n = 11;10) | 0.11 ± 0.01 | | | 0.15 ± 0.01 | |
| donor II (n = 10;9) | 0.13 ± 0.01 | | | 0.142 ± 0.002 | |
| donor III (n = 9;9) | 0.111 ± 0.005 | | | 0.126 ± 0.004 | |
| **total** | **0.1152 ± 0.005** | | | **0.1412 ± 0.0039** | |
| statistical analysis: one-sample t-test | | | | | |
| **Supplementary Figure 2 I** | | | | | |
|  | **relative MT1-MMP signal [%]** | | | | |
| **condition** | **donor I** | **donor II** | **donor III** | | **mean ± SEM** |
| DMSO | 100 | 100 | 100 | | **100 ± 0** |
| wortmannin [1 µM] | 109.8 | 83.02 | 101.26 | | **98.02 ± 7.90** |
| statistical analysis: unpaired t-test | | | | | |

| **Supplementary Figure 3** | | | | |
| --- | --- | --- | --- | --- |
|  | | | | |
| **Supplementary Figure 3A** | | | | |
|  | **KIF16B-YFP vesicle size [µm^2^] ± SEM** | | | |
|  | **DMSO** | | **wortmannin** | |
|  | **ctrl** | **Rab14** | **ctrl** | **Rab14** |
| donor I (n = 10) | 0.10 ± 0.013 | 0.24 ± 0.053 | 0.22 ± 0.049 | 0.27 ± 0.016 |
| donor II (n = 10) | 0.12 ± 0.026 | 0.13 ± 0.020 | 0.22 ± 0.024 | 0.26 ± 0.021 |
| donor III (n = 10) | 0.10 ± 0.017 | 0.08 ± 0.005 | 0.23 ± 0.016 | 0.25 ± 0.027 |
| **total** | 0.11 ± 0.011 | 0.15 ± 0.022 | 0.22 ± 0.018 | 0.26 ± 0.012 |
| statistical analysis: unpaired t-test | | | | |
| **Supplementary Figure 3 B** | | | | |
|  | **KIF16B-YFP vesicle number ± SEM** | | | |
|  | **DMSO** | | **wortmannin** | |
|  | **ctrl** | **Rab14** | **ctrl** | **DMSO** |
| donor I (n = 10) | 60.1 ± 12.42 | 118.6 ± 25.07 | 15.1 ± 2.06 | 33.30 ± 5.99 |
| donor II (n = 10) | 102.7 ± 26.68 | 79.9 ± 18.17 | 21.8 ± 3.86 | 25.8 ± 3.77 |
| donor III (n = 10) | 62.3 ± 5.33 | 75.8 ± 11.08 | 16.5 ± 2.82 | 24.2 ± 2.16 |
| **Total** | 75.03 ± 10.28 | 91.43 ± 11.17 | 17.80 ± 1.76 | 27.77 ± 2.49 |
| statistical analysis: unpaired t-test | | | | |

| **Supplementary Figure 4** | | | | | | | | | | |
| --- | --- | --- | --- | --- | --- | --- | --- | --- | --- | --- |
|  | | | | | | | | | | |
| **Supplementary Figure 2 C** | | | | | | | | | | |
|  | | **ctrl**  (n=9) | | **4x10^4^** (n=12) | | **8x10^4^** (n=12) | | **2x10^5^** (n=11) | | **5x10^5^**  (n=9) |
| **Spheroid area [µm^2^] ± SEM** | | 294,121 ± 13,794 | | 325,311 ± 5,855 | | 318,000 ± 11,078 | | 283,052 ± 17,676 | | 246,207 ± 14,774 |
| statistical analysis: ordinary one-way ANOVA | | | | | | | | | | |
| **Supplementary Figure 2 D** | | | | | | | | | | |
|  | | **ctrl**  (n=9) | | **4x10^4^** (n=12) | | **8x10^4^** (n=12) | | **2x10^5^** (n=11) | | **5x10^5^**  (n=9) |
| **Particle number/ spheroid**  **± SEM** | | 98.67 ± 11.92 | | 104.3 ± 13.6 | | 83.92 ± 14.23 | | 132.3 ± 18.87 | | 225.56 ± 27.2 |
| statistical analysis: ordinary one-way ANOVA | | | | | | | | | | |
| **Supplementary Figure 2 E** | | | | | | | | | | |
|  | | | **expression [%]** | | | | | | | |
| **day** | **condition** | | **donor I** | | **donor II** | | **donor III** | | **mean ± SEM** | |
| 3 | ctrl | | 100 | | 100 | | 100 | | **100 ± 0** | |
|  | KIF16B #2 | | 25.40 | | 21.59 | | 17.25 | | **21.41 ± 2.35** | |
| 4 | ctrl | | 100 | | 100 | | 100 | | **100 ± 0** | |
|  | KIF16B #2 | | 11.43 | | 10.74 | | 6.83 | | **9.66 ± 1.43** | |
| 5 | ctrl | | 100 | | 100 | | 100 | | **100 ± 0** | |
|  | KIF16B #2 | | 11.08 | | 14.95 | | 6.55 | | **10.86 ± 2.43** | |
| 6 | ctrl | | 100 | | 100 | | 100 | | **100 ± 0** | |
|  | KIF16B #2 | | 10.84 | | 7.79 | | 7.38 | | **8.67 ± 1.09** | |
| 7 | ctrl | | 100 | | 100 | | 100 | | **100 ± 0** | |
|  | KIF16B #2 | | 18.52 | | 19.88 | | 13.68 | | **17.36 ± 1.88** | |
| 10 | ctrl | | 100 | | 100 | | 100 | | **100 ± 0** | |
|  | KIF16B #2 | | 23.46 | | 44.41 | | 28.41 | | **32.09 ± 6.32** | |
| statistical analysis: one-sample t-test | | | | | | | | | | |
